# Supplementary material for: Predicting visual function by interpreting a neuronal wiring diagram
Source: Nature. 2024 Oct 2;634(8032):113–23. doi: 10.1038/s41586-024-07953-5 (PMC11446822; doi:10.1038/s41586-024-07953-5)
Supplement: Supplementary file 6 — CRF and ERF predictions for individual TmY4 and TmY9 cells. Analogous to Supplementary Data 3, but for TmY target types. Shown are the top four monosynaptic pathways, the strongest pathway passing through each of the top ten intermediary types (ranking from Extended Data Fig. 7), and the trisynaptic pathway Tm1–TmY–Dm3–TmY (see the section entitled Prediction of spatial normalization). [file 41586_2024_7953_MOESM6_ESM.zip › DataS4/TmY9qΓèÑ/720575940622022209.pdf]

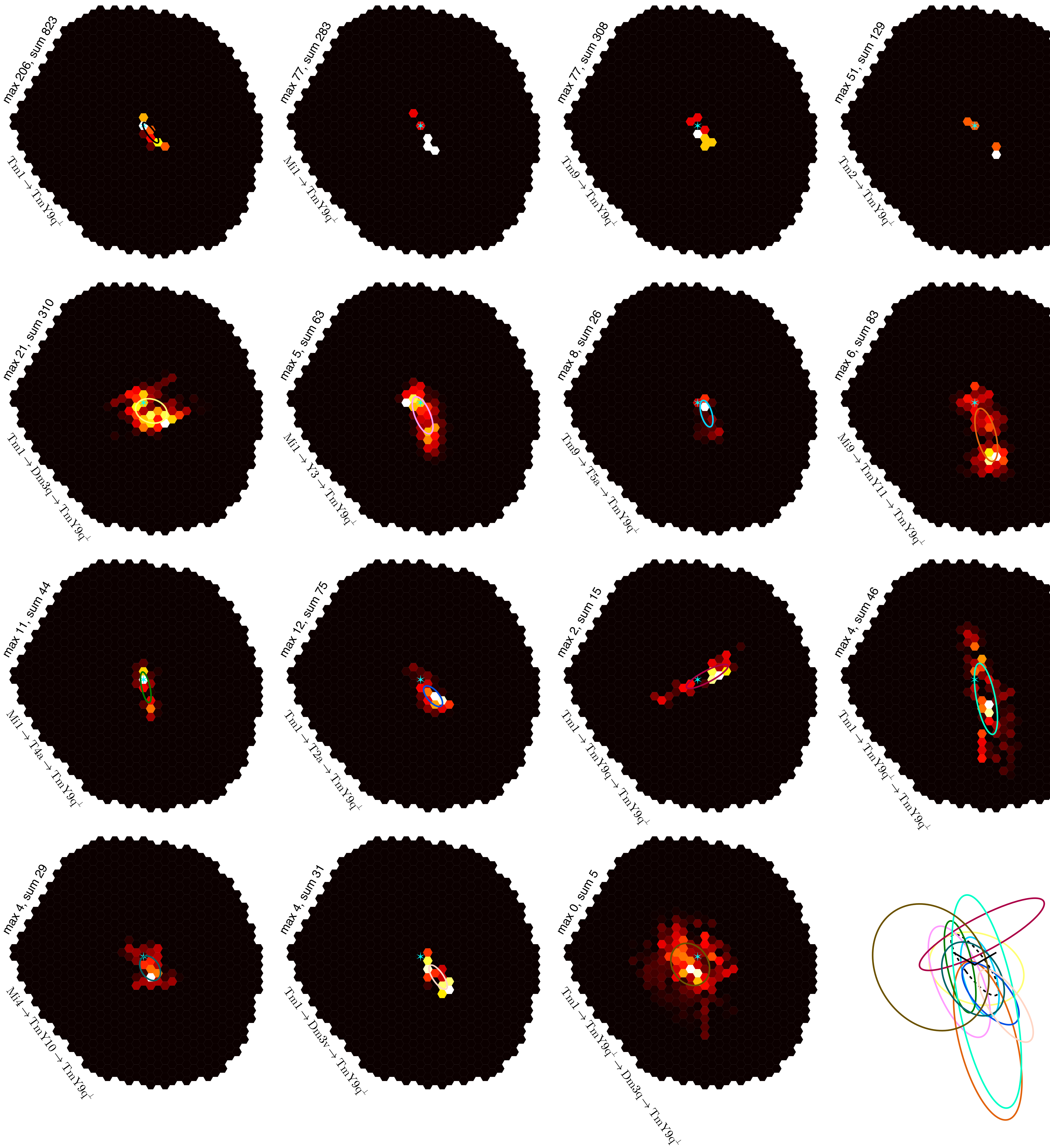
max 206, sum 823
Tm1 → TmY9q⊥
max 77, sum 283
Mi1 → TmY9q⊥
max 77, sum 308
Tm9 → TmY9q⊥
max 51, sum 129
Tm2 → TmY9q⊥
max 21, sum 310
Tm1 → Dm3q → TmY9q⊥
max 5, sum 63
Mi1 → Y3 → TmY9q⊥
max 8, sum 26
Tm9 → T5a → TmY9q⊥
max 6, sum 83
Mi9 → TmY11 → TmY9q⊥
max 11, sum 44
Mi1 → T4a → TmY9q⊥
max 12, sum 75
Tm1 → T2a → TmY9q⊥
max 2, sum 15
Tm1 → TmY9q → TmY9q⊥
max 4, sum 46
Tm1 → TmY9q⊥ → TmY9q⊥
max 4, sum 29
Mi4 → TmY10 → TmY9q⊥
max 4, sum 31
Tm1 → Dm3v → TmY9q⊥
max 0, sum 5
Tm1 → TmY9q⊥ → Dm3q → TmY9q⊥
